# Visualization and Analysis of Air Pollution and Human Health Based on Cluster Analysis: A Bibliometric Review from 2001 to 2021

**DOI:** 10.3390/ijerph191912723

**Published:** 2022-10-05

**Authors:** Diyi Liu, Kun Cheng, Kevin Huang, Hui Ding, Tiantong Xu, Zhenni Chen, Yanqi Sun

**Affiliations:** 1 Zhou Enlai School of Government, Nankai University, Tianjin 300071, China; 2 College of Management and Economy, Tianjin University, Tianjin 300072, China; 3 School of Accounting, Economics and Finance, University of Wollongong, Sydney, NSW 2522, Australia; 4 School of Marxism, Hangzhou Medical College, Hangzhou 310053, China; 5 School of E-Business and Logistics, Beijing Technology and Business University, Beijing 100048, China; 6 School of Economics and Finance, Xi’an Jiaotong University, Xi’an 710061, China; 7 School of Economics and Management, Beijing Institute of Petrochemical Technology, Beijing 102617, China

**Keywords:** air pollution, health, bibliometric, machine learning, social network analysis, co-occurrence network

## Abstract

Bibliometric techniques and social network analysis are employed in this study to evaluate 14,955 papers on air pollution and health that were published from 2001 to 2021. To track the research hotspots, the principle of machine learning is applied in this study to divide 10,212 records of keywords into 96 clusters through OmniViz software. Our findings highlight strong research interests and the practical need to control air pollution to improve human health, as evidenced by an annual growth rate of over 15.8% in the related publications. The cluster analysis showed that clusters C22 (exposure, model, mortality) and C8 (health, environment, risk) are the most popular topics in this field of research. Furthermore, we develop co-occurrence networks based on the cluster analysis results in which a more specific keyword classification was obtained. These key areas include: “Air pollutant source”, “Exposure-Response relationship”, “Public & Occupational Health”, and so on. Future research hotspots are analyzed through characteristics of the cluster groups, including the advancement of health risk assessment techniques, an interdisciplinary approach to quantifying human exposure to air pollution, and strategies in health risk assessment.

## 1. Introduction

The negative effects of air pollution on human health are well documented [1,2]. Harmful air pollutants, such as PM_2.5_, PM_10_, SO_2,_ and NO_X_, escaping into the environment through natural and human activities may adversely affect human health [3]. Air pollution has acute and chronic effects on various systems and organs. These range from upper respiratory tract irritation to chronic respiratory and heart diseases [4], lung cancer [5], childhood acute respiratory infections [6] and adult chronic bronchitis, exacerbating existing cardiopulmonary diseases or asthma attacks [7], and even causing serious mental illness [8]. According to the official statistics of the World Health Organization, the number of people killed by air pollution is as high as 7 million every year, and 9 out of every 10 people in the world still breathe air containing high levels of pollutant concentration [9].

Due to the growing public concern about the adverse health consequences caused by air pollution, an increasing number of publications have examined the correlation between exposure to air pollutants and the incidence and mortality of various diseases [10,11,12,13,14,15,16]. For example, some studies have shown that exposure to particulate matter and ozone in the air is associated with increased mortality and hospitalization due to respiratory and cardiovascular disease [17,18]. Particulate matter is likely to penetrate the lungs and cardiovascular system, leading to diseases such as stroke, heart disease, lung cancer, and chronic obstructive pulmonary disease [19,20]. Nevertheless, most of the publications focusing on specific fields are scattered, and the interrelationship between them is not yet clear. Although many experts have summarized the results of research in this field [21,22,23,24], there are still few studies on the application of bibliometric methods for analysis. So far, there are only two articles that review air pollution and human health with a bibliometric method. Han et al. [25] used bibliometric methods to analyze the relevant literature in the field of health effects caused by PM_2.5_ since 2000. Dhital et al. [26] carried out a bibliometric analysis of 2179 documents published during the last two decades. The limitation of the above two studies is that they mainly focus on the performance of literature from the perspectives of international collaboration, journal distribution, authorship cooperation, etc., which belong to the basic descriptive results. Therefore, in this paper, to further examine the hot research topics, we combine machine learning with bibliometric methods to quantitatively review the literature in this field. The quantitative methods are more conducive to helping us better understand the air pollution and health research landscape.

This paper explores the development of this field through statistical methods from a macro perspective. The primary objective of this study is to give a comprehensive overview of the research publications on air pollution and human health published during the last two decades (2001–2021). The main contributions of this paper are as follows: (1) This study aims to provide a qualitative and quantitative evaluation of the current research progress and trends in air pollution and health research. (2) Some publication characteristics, including countries, funding agencies, journals, and international collaborations, are presented. (3) The study applies the principle of machine learning to divide 10,212 records into 96 clusters and identifies the positional relationship between them. (4) Based on the clustering results, we develop co-occurrence networks and obtain more specific keyword classification results by keyword frequency, relationship, and semantic analysis to obtain future research hotspots.

## 2. Materials and Methods

### 2.1. Data Collection

**Figure 1:**
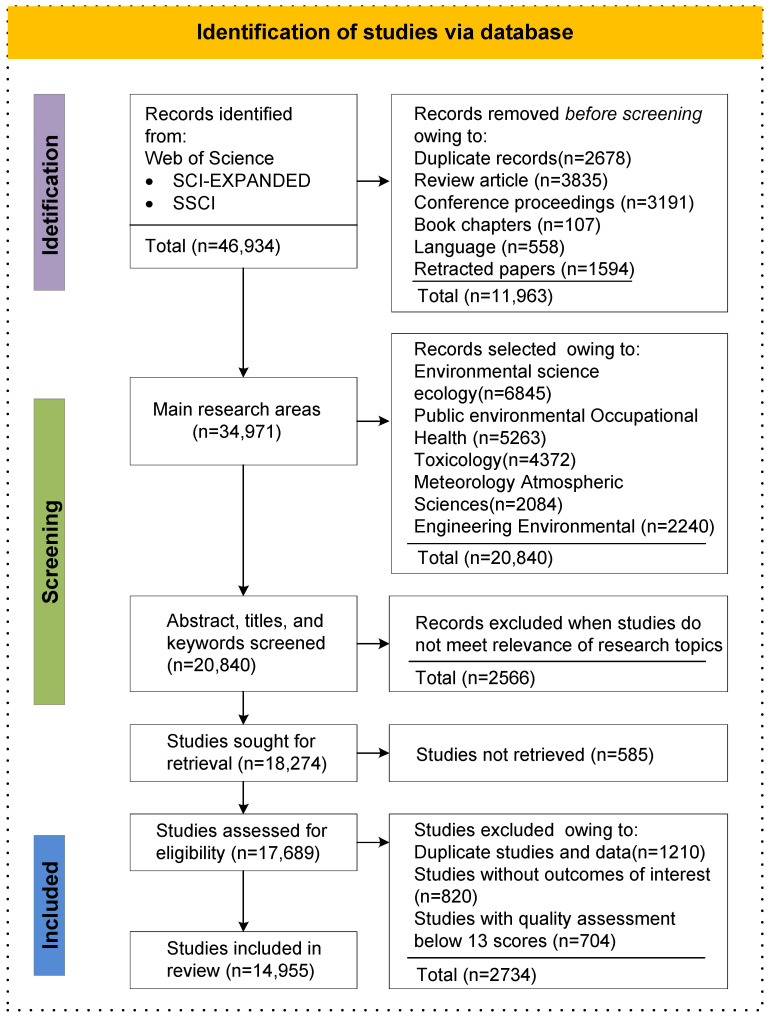
Framework of literature search, analysis, and interpretation.

The Web of Science Core Collection provides a variety of records for each publication, including author information, journals, citations, and institutional affiliations. We mainly search for articles from SCI and SSCI, published in English from 2001 to 2021. The study used keywords (i.e., “air pollution*” or PM_2.5_ or ozone or “particular matter”) and (health or mortality or fatality or death or epidemiology or fitness or morbidity) to search and collect research articles. A total of 46,934 pieces of literature were identified through database searches. After excluding 11,963 pieces of literature of the non-article type and an additional 2566 via screening of the titles and abstracts, finally, 14,955 articles were full-text screened for eligibility (See Figure 1, Figures S1 and Table S1).

In addition, OmniViz (BioWisdom Ltd., Cambridge, UK) was used to extract and cluster keywords from the articles. OmniViz is an advanced visual informatics software package that is designed to provide visualization of digital data, categorical data, genomic sequences, chemical structures, and text documents [27]. It can analyze large data sources through different clustering methods. We used OmniViz to identify important topics and hot research areas. In addition, the clustering method was used to measure the similarity of two records in a high-dimensional space. To achieve data visualization, we used Galaxy and Thememap. Galaxy provides relationships between lots of records, and Thememap identifies the most important topics in the field. Please see Figure S2 in the supplementary file for details on methodology and software.

### 2.2. Impact Factors

The impact factor (IF) and h-index are well-recognized indicators that are closely related to the bibliometric analysis. The IF is a useful indicator to quantify the rank and quality of a journal. The IF of a journal is calculated by dividing the citation count of the current year by the number of published articles in the journal during the previous two years [28]. It is created by the Institute of Scientific Information (ISI). A higher IF usually reflects a journal’s higher quality in various research fields. The h-index means that ‘h’ of one’s total articles are cited at least ‘h’ times. It is a popular indicator to measure the performance of a scientist and has been widely used to evaluate the academic performance of a journal or a country.

### 2.3. Social Network Analysis (SNA)

Social network analysis (SNA) refers to a computable analysis method based on multidisciplinary fusion theories and methods to understand the formation of various human social relationships, behavioral characteristics analysis, and the laws of information transmission. It aims to quantify the network’s structure features and the dynamic interactions among network vertices. Due to the development of social network theory, SNA has been widely used to analyze academic collaboration in different fields [29]. By using a variety of measurement metrics, the contributions from different countries, institutions, and scientists can be evaluated.

In this study, SNA is used to evaluate collaboration among different countries and institutions [30], which includes two steps. The first step is information extraction. The country and institutional information for each author was extracted using BibExcel so that the visualization effect of academic cooperation among different countries can be presented. The second step is to draw a cooperation diagram with the input data from BibExcel using Pajek to visualize their cooperation patterns.

## 3. Results and Discussions

### 3.1. The Performance of Related Publications

**Figure 2:**
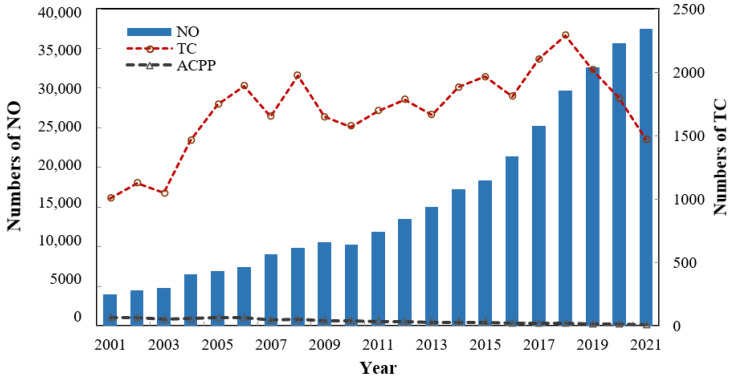
Numbers of the NO, TC, and ACPP during the period of 2001–2021. Notes: NO represents the number of published articles, TC represents the total citations of articles, and ACPP represents the average citation counts of each article.

In recent years, the issues of air pollution have gradually drawn public attention. Our study focuses on the effect of air pollution on health from the perspective of bibliometrics so as to understand the current research status and future research trends. Figure 2 shows the annual number (NO) of articles published between 2001 and 2021, the total number of citations (TC) for the articles, and the average citation count (ACPP) for each article. It can be observed that the NO has grown slowly in the first 12 years and has increased rapidly with a growth rate of more than 9% since 2008. The number of articles published after 2009 accounted for nearly 85% of the total number of published articles. In addition, the TC grew steadily during the first 11 years, peaked in 2008 and 2018, and then gradually declined. Due to the increasing number of published articles, the ACPP showed a downward trend as a whole.

### 3.2. Publication Features of Different Countries

The number of publications reflects the academic strengths and attentions of each country in the field. The top five most productive countries are the United States (6932 publications), China (4350 publications), the United Kingdom (2206 publications), Canada (1035 publications), and Italy (974 publications). These top five countries published a total of over 10,000 articles, accounting for 78.55% of all publications. Among these productive countries, the United States outperforms others in the total number of published articles on air pollution during 2001–2021 [31]. It can be observed that China’s publications have grown rapidly since 2014, with an average growth rate of over 30%. Moreover, the amount of literature published by Chinese scholars gradually approached the USA’s productivity in 2021.

**Figure 3:**
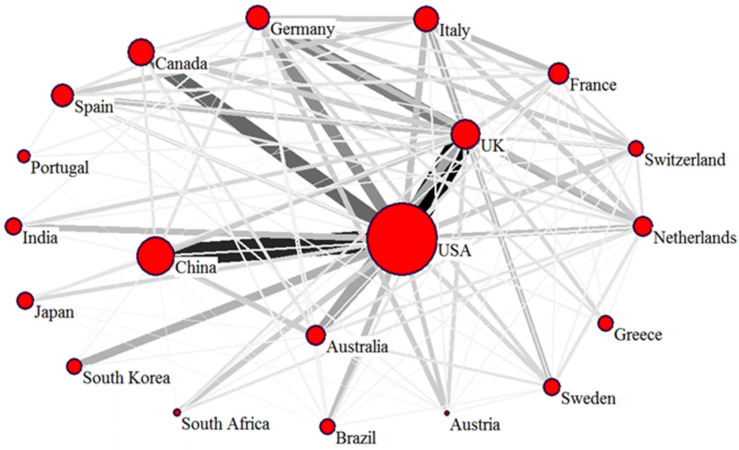
International collaboration according to social network analysis.

We applied SNA to analyze the international collaboration among the 20 most productive countries during the period 2001–2021 (Figure 3). The lines connecting the countries represent their cooperation, and the line thickness indicates the degree of collaboration [32,33]. Collaboration was determined by the affiliations of the co-authors, and all countries or institutes stand to benefit if one publication is a collaborative study [34,35]. These 20 productive countries worked closely with each other, particularly the U.S.A., China, Canada, Germany, and the U.K. The U.S.A. was the center of this collaboration network and the leader of air pollution research in cooperation with the other productive countries. The U.S.A. and China had the closest collaboration, followed by the U.S.A.–U.K., U.S.A.–Canada, and the U.S.A.–Germany.

### 3.3. The Performances of Different Journals

The top 20 most productive journals are shown in Table 1. These productive journals account for 63.4% of the total related publications. In particular, *Atmospheric Environment* is the most productive journal with a count of 1506 (10.07%) articles. Other dominating journals include *Environmental Health Perspectives*, *Science of the Total Environment*, and *Environmental Research*. The IF of the journal is not the only index to reflect the journal’s influence in the field. Therefore, we calculated the average citations to better reflect the journal’s influence. The results showed that *Epidemiology* and *Environmental Health Perspectives* have the highest average citations (86.25 and 81.30), followed by the *American Journal of Epidemiology*, *Occupational and Environmental Medicine*, and *Environmental Science and Technology*.

**Figure 4:**
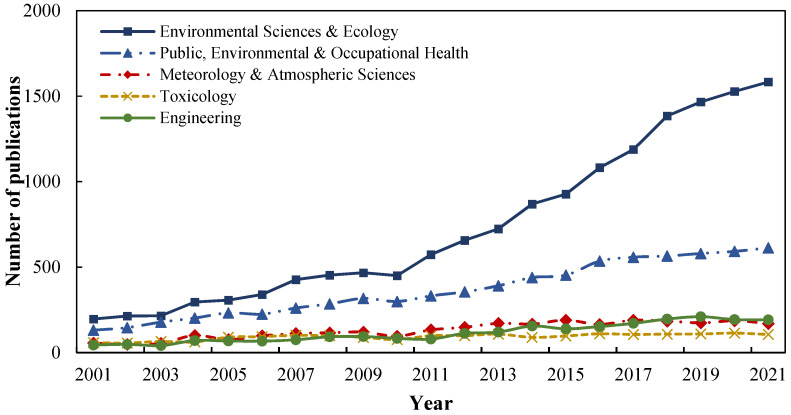
The top 5 research areas of publications.

Furthermore, the field of air pollution and health is typically an interdisciplinary area. According to the statistics (Figure 4), the largest proportion of research areas are in Environmental Sciences and Ecology; Public, Environmental, and Occupational Health; Meteorology and Atmospheric Sciences; and Toxicology, which account for 93.2% of the total number of publications. Among them, the Environmental Sciences and Ecology research area maintains an average growth rate of more than 15%, occupying the most important position.

### 3.4. Institutions’ Performances

The performances of the top 20 most productive institutions are listed in Table 2. Most institutions are from the productive countries shown in the previous section. Among them, twelve institutions are located in the U.S.A and three are from China. Harvard University is the most productive research organization with 1263 publications, followed by the University of California System, the United States Environmental Protection Agency, and the Chinese Academy of Sciences. In the U.S.A., universities and government research institutes, such as the United States Environmental Protection Agency, are the main forces in the field. Additionally, several European countries, such as England, the Netherlands, and Switzerland, have mature experience in air pollution prevention and reducing its negative impact on health. Therefore, it is not surprising to see that 4 institutions in European countries are listed among the top 20 most productive institutions.

### 3.5. Research Hotspots Analysis

#### 3.5.1. Keyword Clustering

**Figure 5:**
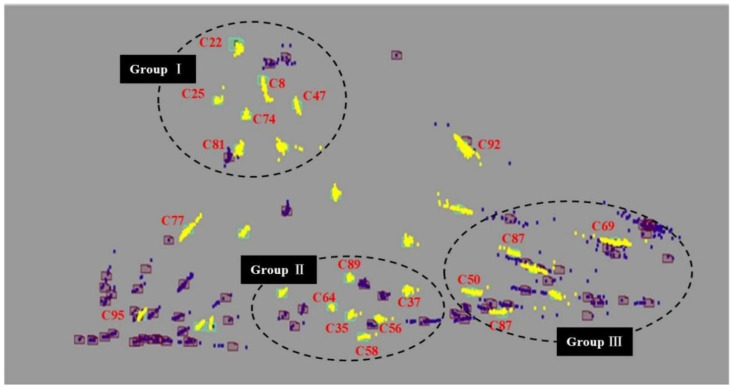
Keywords Galaxy map (records > 50).

A machine learning-based cluster analysis was carried out on the keywords of 14,955 research articles using software named OmniViz. A total of 10,212 records were obtained and divided into 96 clusters. The article selected clusters with more than 50 records, for a total of 29 clusters, as shown in Table 3. Moreover, we also give top 20 frequent keywords list (Table S2). The cluster C22 (exposure, model, mortality) has the most publications with a total of 3629, which accounts for 37.1% of the total. In addition, C8 (health, environment, risk), C92 (health, environment, risk), C35 (exposure, person, particle), C6 (model, ozone, emit), and C64 (aerosol, source, dust) are recorded more than 300 times. These clusters were also usually selected as research topics. In addition, the distance of each cluster in the galaxy map can reflect their correlations. If their locations are closely related to their research relevance, their relevance is very high. On the contrary, these research themes are not strongly related. The clusters of publications recorded with more than 50 instances are shown in Figure 5 and marked as yellow. It can be concluded that C8, C47, C81, and C25 are closed to each other and concentrated at the top of the galaxy map. This indicates that keywords such as “environment, health, and risk” are often associated with “exposure, chronic, disease, heart,” and so on. Similarly, located in the middle and lower parts of the galaxy map, C89, C64, C35, C58, and C56 are also closely linked. This suggests that the relationships between the keywords of “particle, ozone, dust”, and other related pollutants and “aerosol, concentration, source” are very close. In addition, clusters C8 and C92 show the same term labels, both of which are “health, environment, risk”. The number of records is above 500. However, they are from different collections of publications at different positions on the galaxy map (Figure 5). Each pollutant has different sources and measurement methods at the pollutant level [36]. The health level involves various diseases, and the related model methods are often employed in related research [37].

**Figure 6:**
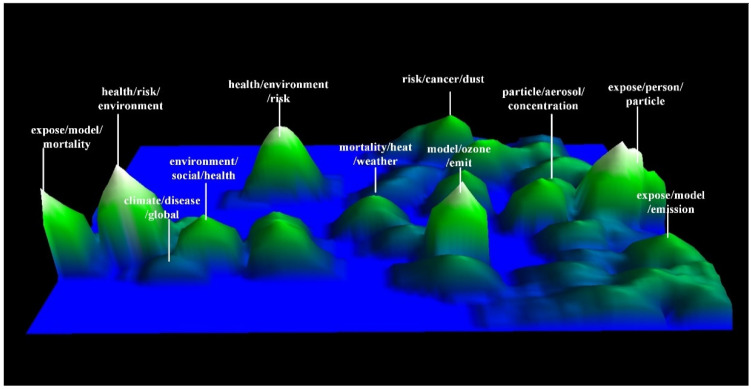
Theme map of keywords.

In addition, as shown in Figure 6, a theme map can identify the main topics in the research field of air pollution and health, which is an effective complement to the Galaxy visualization. The height of the peak depends on the intensity of the topic and the concentration of information at that location. It can be observed that the four highest peaks are: “health, environment, risk”, “exposure, model, mortality”, “exposure, ozone, person”, and “model, ozone, emit”. The results are very similar to the cluster analysis; however, there are some differences in the height order. The main reason is that, although C22 has the most records, it is not closely related to the surrounding clusters. The cluster C8 is more intensive at this position, resulting in a stronger theme, which led to the highest peak. Compared with others, the peaks around “exposure, person, particle” are significantly denser. There are more valleys surrounding the rest of the peaks, which suggests that such research is highly relevant and often involves an interdisciplinary approach.

#### 3.5.2. Relationship of Keywords among Different Groups

**Figure 7:**
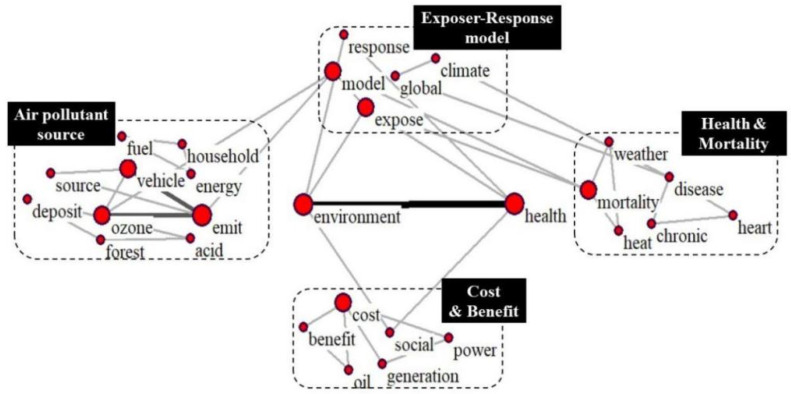
Group I research topics.

Through keyword clustering, 10,212 records were divided into 96 clusters. In the keywords Galaxy map (Figure 7), we can roughly divide them into three groups, i.e., Group I, Group II, and Group III, based on the positional relationships of different clusters. Although the clusters located at the lower left of the Galaxy map are very dense, most of these clusters appear less than 50 times and, therefore, will not be analyzed further. Within each group, we develop co-occurrence networks and obtain more specific keyword classifications based on keyword frequency, relationship, and semantic analysis. The research hotspots are analyzed through the characteristics of three groups.

(1) Group I

The impact of the deterioration of the ecological environment, especially of the air quality, on human health has received a growing level of attention. The damage to health caused by air pollution further increases the degree of health inequalities among groups of different income levels [38,39]. As shown in Figure 7, “environment and health” are recognized as the central keywords of Group I because of their high frequencies and close relationships with other research topics. Focusing on two central keywords, we identified four relevant research areas, i.e., “Air pollutant source”, “Exposure–Response relationship”, “Health & Mortality”, and “Cost & Benefit”. In terms of air pollutant sources, outdoor sources often refer to the cluster C37 (Emit, vehicle, industry), mainly including industry and vehicle emissions. Household pollution sources, such as the keyword clusters C77 (Fuel, energy, household) and C20 (Standard, management, ventilation), are often combined with specific issues, such as fuel burning, use of building materials, chemicals, and ventilation in household activities [40]. Most air pollutants monitored by remote sensing technology are always assessed by the exposure-response function [41], such as the clusters C6 (Model, Ozone, emit) and C89 (Particle, aerosol, concentration), to reflect such research trends. In addition, some studies have stated that air pollution can cause increased morbidity, including heart disease and chronic diseases [42]. And in Table S3, we also summarized highly co-cited documents among research clusters in Clusters I.

(2) Group II

**Figure 8:**
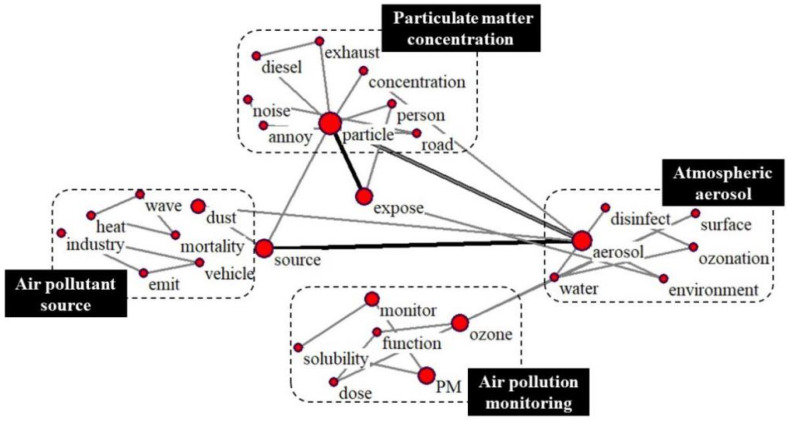
Group II research topics.

As shown in Figure 8, “exposure” is recognized as a core keyword, and three keywords with high frequency are closely related (i.e., particle, aerosol, source). With these four keywords as the core, four relevant research areas have been formed, namely “Air pollution source”, “Air pollution monitoring”, “Particulate matter concentration”, and “Atmospheric aerosol”. The current methods of air pollution control focus on source management, so identifying air pollution sources is still an important research area [43].Moreover, dust in cities can also increase the short-term mortality of vulnerable populations, such as extreme dust episodes in high-density Asian cities [44]. In order to formulate reasonable air pollution control policies, real-time monitoring of air quality becomes more important. In addition to common air monitoring stations [45], some studies suggest that air quality monitoring can be performed in innovative ways, such as through social media [46] and mobile sensors [47]. Though PM and ozone are the main monitored pollutants, related studies have gradually evolved from single-pollutant to multi-pollutant collaborative studies, such as ozone, particle, carbon monoxide, PM_2.5_, PM_10_, etc. [48]. As shown in Figure 8, there is a strong relationship between “particle” and “aerosol”. Studies have found that severe haze pollution incidents were mainly caused by the formation of secondary aerosols. Moreover, in Table S4, we also summarized highly co-cited documents among research clusters in Clusters II.

(3) Group III

**Figure 9:**
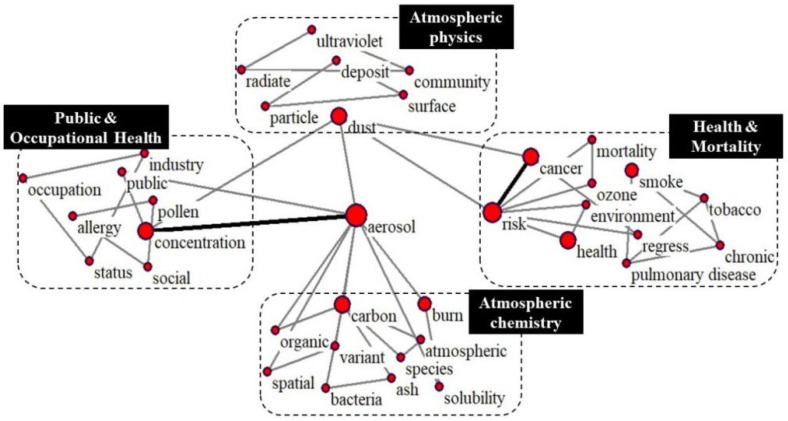
Group III research topics.

As shown in Figure 9, “aerosol” is the central word in this section. Around the central keyword, four related research areas can be identified, including “Atmospheric physics”, “Atmospheric chemistry”, “Health & Mortality”, and “Public & Occupational Health”. Atmospheric physics and atmospheric chemistry are the basic disciplines in related research. They can explain the formation and transmission mechanisms of atmospheric pollutants and provide a theoretical basis for controlling air pollution [49]. The research field “Health & Mortality” was once again emphasized in the clustering results. Compared with Group I, this part has more diseases mentioned. The measurement of health risks from the mortality index [50] gradually concentrated on the incidence of specific diseases, including cancer, heart disease, respiratory diseases, and so on. Further, based on the complexity of multi-pollutant collaborative research [51], studies must develop more targeted evaluation models and apply new model fusion methods, i.e., the air pollution mortality/incidence risk (Ri-MAP) model [52], comprehensive health risk index, exposure–response coefficient [53], CMAQ/GCAM evaluation model [54], and so on. We are pleasantly surprised to find that public and occupational health research is further valued in Group III. Some studies currently classify occupational characteristics and social status to assess the health effects of air pollution on different groups [55]. For example, the socioeconomic status of parents, including education, income, and living area, has an impact on children’s health, which suggests that health can play an important role in the intergenerational transmission of economic status [56]. In addition, the highly co-cited documents among research clusters in Cluster II have been summarized in Table S5.

## 4. Conclusions

### 4.1. Summary

This study conducted a bibliometric analysis of 14,955 articles on air pollution and health from 2001 to 2021. In the past two decades, the most productive country, the most productive institution, and the most productive journal are the United States, Harvard University, and *Atmospheric Environment*, respectively. We used OmniViz to cluster the keywords of 10,212 records, and the results show that the clusters with the most occurrences were “Exposure, model, mortality”, “Health, environment, risk”, and “Model, Ozone, emit”. By observing the Thememap and Galaxy visualization results, additional popular topics in the study include “Health, environment, risk” and “Exposure, person, particle”. Based on the clustering results, we developed co-occurrence networks and obtained more specific keyword classification results according to keyword frequency, relationship, and semantic analysis. We have identified the most influential areas, such as “Air pollutant source”, “Exposure–Response relationship”, “Public & Occupational Health”, and so on. The research hotspots are analyzed through the characteristics of three groups of clusters. Indeed, this paper provides a qualitative and quantitative evaluation of the current research progress and trends in air pollution and health research.

### 4.2. Limitations and Future Research Directions

Nonetheless, although bibliometric analysis is an effective method for reviewing the literature, it is not without limitations. First, the bibliometric data from the Web of Science (including SCI-EXPAND and SSCI) are not produced exclusively for analysis, thus the data may contain errors, wherein the presence of errors is bound to influence any analysis performed using such data. Thus, to mitigate errors, we have carefully cleaned the bibliometric data that we searched. For example, we remove duplicates and erroneous entries. Second, the nature of the bibliometric method is in itself a limitation. We noticed that the qualitative assertions of bibliometrics can be subjective given that bibliometric analysis is quantitative in nature, whereas the relationship between qualitative and quantitative results is often unclear. To solve these problems, we tried to combine machine learning and the bibliometric method to make a more accurate results analysis, but there is still room for method improvement. Third, bibliometric studies can only offer a certain period forecast of the research field, and thus scholars should avoid making overly ambitious assertions in their research field. Notwithstanding these limitations, the bibliometric method can help us to overcome the fear of large bibliometric datasets and to pursue retrospectives of air pollution and human health. Indeed, the bibliometric methods can not only facilitate knowledge in this field but also help us to better understand the research trend. We take a short yet significant step in that direction.

Based on the bibliometric analysis, three main future directions of air pollution and health are identified in this study. The first future research direction is the advancement of health risk assessment techniques. At present, the air monitoring network does not take into account health factors such as changes in air pollution components, public medical data, and population activity patterns [57]. There is a lack of dynamic data to support the health risk assessment as the monitoring network of air pollution’s health impact is far from mature [58]. Health risk assessment techniques currently face the challenge of transformation from qualitative research to quantitative research. Future research can be conducted to examine the route and trajectory of pollutant exposure to determine the actual intake and intake coefficient of air pollutants [59]. Subsequently, sophisticated time-activity patterns and individual exposure monitoring techniques should be employed more widely to determine accurate exposure doses [60]. More suitable biological targets should be identified to establish a quantitative relationship between the growth in the concentration of pollutants and the increase in mortality and disease prevalence.

The second future research direction is an interdisciplinary approach to quantifying human exposure to air pollution. Quantifying human exposure to air pollutants is a challenging task [61]. Exposure results from multifaceted relationships and interactions between environmental and human systems, adding complexity to the assessment process [62]. For assessment of the health risks of air pollution, related studies might evolve from epidemiology research to toxicology research. From the perspective of environmental toxicology, future studies can be undertaken on the biological mechanism of the bioavailability and toxicity of particulate matter. In order to establish more accurate quantitative models, real-time air pollution health risks can be described using mobile air pollution monitoring techniques, meteorological information, and land use information [61]. Air quality changes and mobile monitoring allow relevant departments to respond to these changes quickly. Consequently, the threshold for the impact of different pollutants on human health can be determined more accurately.

The third future research direction is management strategies in health risk assessment [63]. Proven effective management strategies include the establishment and enforcement of air standards, reduction in emissions from coal-fired power plants and other stationary sources, banning of the use of polluting fuels in urban centers, improvements to access to public transportation, and so on [64]. Future air pollution prevention strategies will emphasize integration and cooperation. Air pollution control in some key areas should pay attention to regional and departmental cooperation. Policy boundaries will become increasingly blurred, and mandatory regulatory policies may also need to incorporate economic incentives. A simple economic incentive policy will have a small audience and need to be combined with other policies to innovate the current policy tool system. Risk management strategies will shift from simple restrictions and prohibitions to more flexible multi-policy coordination.

## Figures and Tables

**Table 1:** The top 20 most productive journals.

Journal Titles	Records	Percentage	IF2022 ^1^	Average Citations
International Journal of Environmental Research and Human Health	1506	10.07%	4.61	15.70
Science of the Total Environment	1415	9.46%	10.75	26.33
Atmospheric Environment	1240	8.29%	5.75	35.20
Environmental Research	1110	7.42%	8.43	28.85
Environmental Health Perspectives	971	6.49%	11.03	81.30
Environment International	861	5.75%	9.62	23.44
Environmental Pollution	731	4.88%	9.98	25.22
Environmental Science and Technology	632	4.22%	11.35	46.15
Environmental Science and Pollution Research	538	3.59%	5.19	11.72
Journal of the Air and Waste Management Association	510	3.41%	2.63	35.67
Environmental Science and Pollution Research	486	3.24%	5.19	12.56
Environmental Health	435	2.90%	7.12	20.90
Atmospheric Chemistry and Physics	407	2.72%	7.19	31.64
Air Quality Atmosphere and Health	359	2.40%	5.80	13.68
Journal of Exposure Science and Environmental Epidemiology	336	2.24%	6.37	22.73
Epidemiology	321	2.15%	4.99	86.25
Journal of Toxicology and Environmental Health-Part A-Current Issues	278	1.85%	2.71	24.15
Aerosol and Air Quality Research	266	1.77%	2.59	10.33
Environmental Monitoring and Assessment	227	1.52%	1.80	13.57
Occupational and Environmental Medicine	202	1.35%	3.97	50.23
American Journal of Epidemiology	198	1.32%	4.32	67.28

^1^ Note: IF2022 represents the published impact factor for 2022. The impact factor is a measure of the importance of a journal. The impact factor is calculated by dividing the number of times the articles are cited in the last two years by the total number of publications in those two years.

**Table 2:** The top 20 most productive institutions.

Rank	Institutions	Country	Records
1	Harvard University	USA	1263
2	University of California System	USA	1230
3	United States Environmental Protection Agency	USA	1205
4	Chinese Academy of Sciences	China	1183
5	University of California Berkeley	USA	1005
6	University of Northern California	USA	956
7	University of London	UK	901
8	Peking University	China	874
9	Helmholtz Association	Germany	856
10	University of Washington	USA	764
11	University of Washington—Seattle	USA	732
12	Utrecht University	The Netherlands	724
13	Health Canada	Canada	683
14	University of North Carolina at Chapel Hill	USA	664
15	University of Southern California	USA	628
16	Imperial College London	UK	540
17	Emory University	USA	529
18	Johns Hopkins University	USA	487
19	Fudan University	China	430
20	Columbia University	USA	425

**Table 3:** The selected clusters (records > 50) by OmniViz analysis.

No.	Records	Major Terms	No.	Records	Major Terms
C6	456	Model, ozone, emit	C56	242	Ozone, water, surface
C8	722	Health, environment, risk	C58	181	Particle, source, aerosol
C17	76	Carbon, organic carbon, aerosol	C59	93	Model, exposure, process
C20	87	Standard, management, ventilation	C64	336	Aerosol, source, dust
C22	3629	Exposure, model, mortality	C69	162	Risk, cancer, dust
C23	107	Mortality, heat, weather	C74	157	Environment, social, health
C24	69	Aerosol, concentration, process	C77	89	Fuel, energy, household
C25	52	Climate, disease, global	C81	136	Disease, heart, chronic
C32	121	Mortality, ozone, risk	C84	182	Sense, aerosol, source
C35	482	Exposure, person, particle	C85	97	Emit, vehicle, ozone
C37	68	Emit, vehicle, industry	C87	230	Species, atmospheric, carbon
C43	115	Heat, wave, mortality	C89	172	Particle, aerosol, concentration
C47	54	Health, exposure, environment	C92	537	Health, environment, risk
C48	121	Environment, exposure, particle	C95	56	Dioxide, ozone, carbon
C50	58	Smoke, tobacco, chronic obstructive pulmonary disease			

## Data Availability

The original contributions presented in the study are included in the supplementary material. Further inquiries can be directed to the corresponding author.

## Supplementary Materials

The following supporting information can be downloaded at: https://www.mdpi.com/article/10.3390/ijerph191912723/s1. Figure S1: Framework of literature search, analysis, and interpretation. Note: Science Citation Index (SCI); Social Science Citation Index (SSCI). Figure S2: Methodology and software support. Table S1: Keywords used in the search and results. Table S2: The trend of 15 most frequent keywords during 2001–2021. Table S3: Highly co-cited documents among research clusters in Cluster I. Table S4: Highly co-cited documents among research clusters in Cluster II. Table S5: Highly co-cited documents among research clusters in Cluster III.

## References

[1] Cao J., Xu H., Xu Q., Chen B., Kan H. (2012). Fine Particulate Matter Constituents and Cardiopulmonary Mortality in a Heavily Polluted Chinese City. Environ. Health Perspect..

[2] Lelieveld J., Evans J.S., Fnais M., Giannadaki D., Pozzer A. (2015). The contribution of outdoor air pollution sources to premature mortality on a global scale. Nature.

[3] Crouse D.L., Peters P.A., Hystad P., Brook J.R., van Donkelaar A., Martin R.V., Villeneuve P.J., Jerrett M., Goldberg M.S., Pope C.A. (2015). Ambient PM_2.5_, O_3_, and NO_2_ Exposures and Associations with Mortality over 16 Years of Follow-Up in the Canadian Census Health and Environment Cohort (CanCHEC). Environ. Health Perspect..

[4] Peng R.D., Bell M.L., Geyh A.S., McDermott A., Zeger S.L., Samet J.M., Dominici F. (2009). Emergency Admissions for Cardiovascular and Respiratory Diseases and the Chemical Composition of Fine Particle Air Pollution. Environ. Health Perspect..

[5] Hamra G.B., Guha N., Cohen A., Laden F., Raaschou-Nielsen O., Samet J.M., Vineis P., Forastiere F., Saldiva P., Yorifuji T. (2014). Outdoor Particulate Matter Exposure and Lung Cancer: A Systematic Review and Meta-Analysis. Environ. Health Perspect..

[6] Clark N.A., Demers P.A., Karr C.J., Koehoorn M., Lencar C., Tamburic L., Brauer M. (2010). Effect of Early Life Exposure to Air Pollution on Development of Childhood Asthma. Environ. Health Perspect..

[7] McConnell R., Islam T., Shankardass K., Jerrett M., Lurmann F., Gilliland F., Gauderman J., Avol E., Künzli N., Yao L. (2010). Childhood Incident Asthma and Traffic-Related Air Pollution at Home and School. Environ. Health Perspect..

[8] Kampa M., Castanas E. (2008). Human health effects of air pollution. Environ. Pollut..

[9] Schraufnagel D.E., Balmes J.R., Cowl C.T., De Matteis S., Jung S.-H., Mortimer K., Perez-Padilla R., Rice M.B., Riojas-Rodriguez H., Sood A. (2019). Air Pollution and Noncommunicable Diseases: A Review by the Forum of International Respiratory Societies’ Environmental Committee, Part 1: The Damaging Effects of Air Pollution. Chest.

[10] Tagaris E., Liao K.-J., DeLucia A.J., Deck L., Amar P., Russell A.G. (2009). Potential impact of climate change on air pollution-related human health effects. Environ. Sci. Techol..

[11] Brunekreef B., Holgate S.T. (2002). Air pollution and health. Lancet.

[12] Gu H., Cao Y., Elahi E., Jha S.K. (2019). Human health damages related to air pollution in China. Environ. Sci. Pollut. Res..

[13] Harrison R.M., Masiol M., Vardoulakis S. (2015). Civil aviation, air pollution and human health. Environ. Res. Lett..

[14] Ghose M.K., Paul R., Banerjee R.K. (2005). Assessment of the status of urban air pollution and its impact on human health in the city of Kolkata. Environ. Monit. Assess..

[15] Gu H., Yan W., Elahi E., Cao Y. (2020). Air pollution risks human mental health: An implication of two-stages least squares estimation of interaction effects. Environ. Sci. Pollut. Res..

[16] Barman S.C., Kumar N., Singh R., Kisku G.C., Khan A.H., Kidwai M.M., Murthy R.C., Negi M.P.S., Pandey P., Verma A.K. (2010). Assessment of urban air pollution and it’s probable health impact. J. Environ. Biol..

[17] Dockery D.W. (2001). Epidemiologic evidence of cardiovascular effects of particulate air pollution. Environ. Health Perspect..

[18] Huang J., Pan X., Guo X., Li G. (2018). Health impact of China’s Air Pollution Prevention and Control Action Plan: An analysis of national air quality monitoring and mortality data. Lancet Planet. Health.

[19] Adhikari A., Yin J. (2020). Short-term effects of ambient ozone, PM_2.5_, and meteorological factors on COVID-19 confirmed cases and deaths in Queens, New York. Int. J. Environ. Res. Public Health.

[20] Man S.P., Van Eeden S., Sin D.D. (2012). Vascular risk in chronic obstructive pulmonary disease: Role of inflammation and other mediators. Can. J. Cardiol..

[21] Afroz R., Hassan M.N., Ibrahim N.A. (2003). Review of air pollution and health impacts in Malaysi. Environ. Res..

[22] Rückerl R., Schneider A., Breitner S., Cyrys J., Peters A. (2011). Health effects of particulate air pollution: A review of epidemiological evidence. Inhal. Toxicol..

[23] Qiu Y., Zuo S., Yu Z., Zhan Y., Ren Y. (2021). Discovering the effects of integrated green space air regulation on human health: A bibliometric and meta-analysis. Ecol. Indic..

[24] Noël C., Vanroelen C., Gadeyne S. (2021). Qualitative research about public health risk perceptions on ambient air pollution. A review study. SSM-Popul. Health.

[25] Han M., Yang F., Sun H. (2021). A bibliometric and visualized analysis of research progress and frontiers on health effects caused by PM_2.5_. Environ. Sci. Pollut. Res..

[26] Dhital S., Rupakheti D. (2019). Bibliometric analysis of global research on air pollution and human health: 1998–2017. Environ. Sci. Pollut. Res..

[27] Yang Y., Akers L., Klose T., Yang C.B. (2008). Text mining and visualization tools—Impressions of emerging capabilities. World Pat. Inf..

[28] Zhang S., Mao G., Crittenden J., Liu X., Du H. (2017). Groundwater remediation from the past to the future: A bibliometric analysis. Water Res..

[29] Phelps C.C., Heidl R.A., Wadhwa A. (2012). Knowledge, Networks, and Knowledge Networks: A Review and Research Agenda. J. Manag..

[30] Mao G., Liu X., Du H., Zuo J., Wang L. (2015). Way forward for alternative energy research: A bibliometric analysis during 1994–2013. Renew. Sustain. Energy Rev..

[31] Correia A.W., Pope C.A., Dockery D.W., Wang Y., Ezzati M., Dominici F. (2013). Effect of Air Pollution Control on Life Expectancy in the United States An Analysis of 545 US Counties for the Period from 2001 to 2007. Epidemiology.

[32] Du H., Liu D., Lu Z., Crittenden J., Mao G., Wang S., Zou H. (2019). Research development on sustainable urban infrastructure from 1991 to 2017: A bibliometric analysis to inform future innovations. Earth’s Future.

[33] Cobo M.J., Martínez M.Á., Gutiérrez-Salcedo M., Fujita H., Herrera-Viedma E. (2015). 25 years at knowledge-based systems: A bibliometric analysis. Knowl. Based Syst..

[34] Hood W., Wilson C. (2001). The literature of bibliometrics, scientometrics, and informetrics. Scientometrics.

[35] Wu Y., Chen J., Fang H., Wan Y. (2020). Intimate partner violence: A bibliometric review of literature. Int. J. Environ. Res. Public Health.

[36] Kloog I., Ridgway B., Koutrakis P., Coull B.A., Schwartz J.D. (2013). Long- and Short-Term Exposure to PM_2.5_ and Mortality: Using Novel Exposure Models. Epidemiology.

[37] Martens W.J. (1998). Health impacts of climate change and ozone depletion: An ecoepidemiologic modeling approach. Environ. Health Perspect..

[38] Liu F., Zheng M., Wang M. (2020). Does air pollution aggravate income inequality in China? An empirical analysis based on the view of health. J. Clean. Prod..

[39] Yang T., Liu W. (2018). Does air pollution affect public health and health inequality? Empirical evidence from China. J. Clean. Prod..

[40] Prud’homme G., Dobbin N.A., Sun L., Burnett R.T., Martin R.V., Davidson A., Cakmak S., Villeneuve P.J., Lamsal L.N., van Donkelaar A. (2013). Comparison of remote sensing and fixed-site monitoring approaches for examining air pollution and health in a national study population. Atmos. Environ..

[41] To T., Zhu J., Villeneuve P.J., Simatovic J., Feldman L., Gao C., Williams D., Chen H., Weichenthal S., Wall C. (2015). Chronic disease prevalence in women and air pollution—A 30-year longitudinal cohort study. Environ. Int..

[42] Yang J., Zhang B. (2018). Air pollution and healthcare expenditure: Implication for the benefit of air pollution control in China. Environ. Int..

[43] Huang R.-J., Zhang Y., Bozzetti C., Ho K.-F., Cao J.-J., Han Y., Daellenbach K.R., Slowik J.G., Platt S.M., Canonaco F. (2014). High secondary aerosol contribution to particulate pollution during haze events in China. Nature.

[44] Ho H.C., Wong M.S., Yang L., Chan T.-C., Bilal M. (2018). Influences of socioeconomic vulnerability and intra-urban air pollution exposure on short-term mortality during extreme dust events. Environ. Pollut..

[45] Knox A., Mykhaylova N., Evans G.J., Lee C.J., Karney B., Brook J.R. (2013). The expanding scope of air pollution monitoring can facilitate sustainable development. Sci. Total Environ..

[46] Hswen Y., Qin Q., Brownstein J.S., Hawkins J.B. (2019). Feasibility of using social media to monitor outdoor air pollution in London, England. Prev. Med..

[47] Zaldei A., Camilli F., De Filippis T., Di Gennaro F., Di Lonardo S., Dini F., Gioli B., Gualtieri G., Matese A., Nunziati W. (2017). An integrated low-cost road traffic and air pollution monitoring platform for next citizen observatories. Transp. Res. Procedia.

[48] Chan C.K., Yao X. (2008). Air pollution in mega cities in China. Atmos. Environ..

[49] Ma J., Xu X., Zhao C., Yan P. (2012). A review of atmospheric chemistry research in China: Photochemical smog, haze pollution, and gas-aerosol interactions. Adv. Atmos. Sci..

[50] Lim C.C., Hayes R.B., Ahn J., Shao Y., Silverman D.T., Jones R.R., Garcia C., Thurston G.D. (2018). Association between long-term exposure to ambient air pollution and diabetes mortality in the US. Environ. Res..

[51] Wesson K., Fann N., Morris M., Fox T., Hubbell B. (2010). A multi–pollutant, risk–based approach to air quality management: Case study for Detroit. Atmos. Pollut. Res..

[52] Khaniabadi Y.O., Sicard P., Takdastan A., Hopke P.K., Taiwo A.M., Khaniabadi F.O., De Marco A., Daryanoosh M. (2019). Mortality and morbidity due to ambient air pollution in Iran. Clin. Epidemiol. Glob. Health.

[53] Cox L.A. (2018). Effects of exposure estimation errors on estimated exposure-response relations for PM_2.5_. Environ. Res..

[54] Shi W., Ou Y., Smith S.J., Ledna C.M., Nolte C.G., Loughlin D.H. (2017). Projecting state-level air pollutant emissions using an integrated assessment model: GCAM-USA. Appl. Energy.

[55] Mbelambela E.P., Hirota R., Eitoku M., Muchanga S.M.J., Kiyosawa H., Yasumitsu-Lovell K., Lawanga O.L., Suganuma N. (2017). Occupation exposed to road-traffic emissions and respiratory health among Congolese transit workers, particularly bus conductors, in Kinshasa: A cross-sectional study. Environ. Health Prev. Med..

[56] Currie J. (2009). Healthy, Wealthy, and Wise: Socioeconomic Status, Poor Health in Childhood, and Human Capital Development. J. Econ. Lit..

[57] Adams M.D., Kanaroglou P.S. (2016). Mapping real-time air pollution health risk for environmental management: Combining mobile and stationary air pollution monitoring with neural network models. J. Environ. Manag..

[58] Li J., Zhu Y., Kelly J.T., Jang C.J., Wang S., Hanna A., Xing J., Lin C.-J., Long S., Yu L. (2019). Health benefit assessment of PM_2.5_ reduction in Pearl River Delta region of China using a model-monitor data fusion approach. J. Environ. Manag..

[59] Johns D.O., Stanek L.W., Walker K., Benromdhane S., Hubbell B., Ross M., Devlin R.B., Costa D.L., Greenbaum D.S. (2012). Practical Advancement of Multipollutant Scientific and Risk Assessment Approaches for Ambient Air Pollution. Environ. Health Perspect..

[60] Park Y.M., Kwan M.-P. (2017). Individual exposure estimates may be erroneous when spatiotemporal variability of air pollution and human mobility are ignored. Health Place.

[61] Heroux M.-E., Anderson H.R., Atkinson R., Brunekreef B., Cohen A., Forastiere F., Hurley F., Katsouyanni K., Krewski D., Krzyzanowski M. (2015). Quantifying the health impacts of ambient air pollutants: Recommendations of a WHO/Europe project. Int. J. Public Health.

[62] Steinle S., Reis S., Sabel C.E. (2013). Quantifying human exposure to air pollution—Moving from static monitoring to spatio-temporally resolved personal exposure assessment. Sci. Total Environ..

[63] Wang S., Hao J. (2012). Air quality management in China: Issues, challenges, and options. J. Environ. Sci..

[64] Cai S., Ma Q., Wang S., Zhao B., Brauer M., Cohen A., Martin R.V., Zhang Q., Li Q., Wang Y. (2018). Impact of air pollution control policies on future PM_2.5_ concentrations and their source contributions in China. J. Environ. Manag..

