# Supplementary material for: Visualization and Analysis of Air Pollution and Human Health Based on Cluster Analysis: A Bibliometric Review from 2001 to 2021
Source: Int J Environ Res Public Health. 2022 Oct 5;19(19):12723. doi: 10.3390/ijerph191912723 (PMC9566718; doi:10.3390/ijerph191912723)
Supplement: Supplementary file 1 [file ijerph-19-12723-s001.zip › ijerph-1856402-supplementary.pdf]

Supplementary File

1. Research Framework

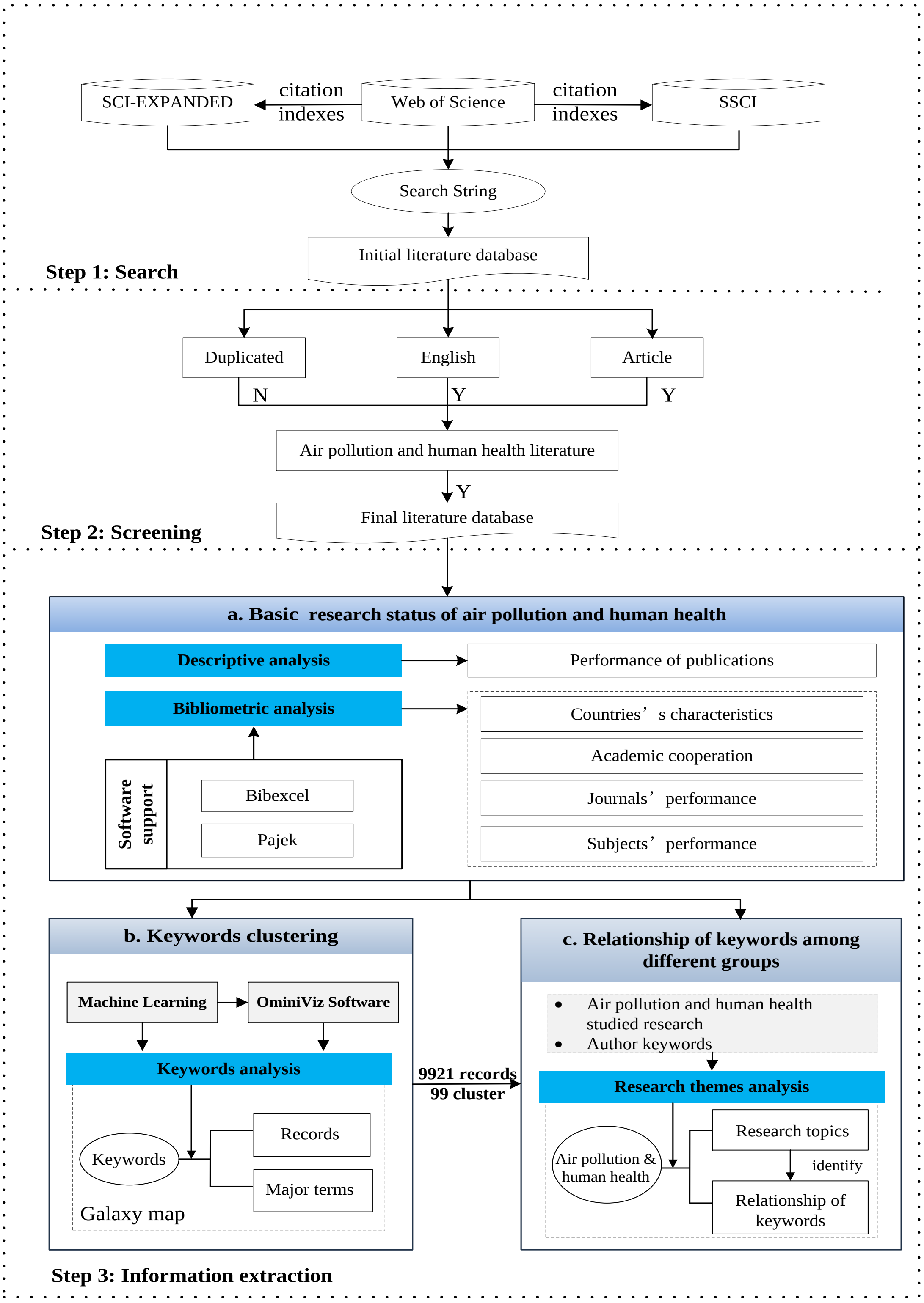

Figure S1 Framework of literature search, analysis, and interpretation. Note: Science Citation Index (SCI); Social Science Citation Index (SSCI).

2. Keywords used in the search

Table S1 Keywords used in the search and results

Search Query	Keywords in Search Process
#1	TS=(“ air pollu*” OR “air quality” OR “atmospheric pollution” OR “$PM_{2.5}$” OR “ozone” OR “particular matter”)
#2	TS=(“health” OR “mortality” OR “fatality” OR “death” OR “epidemiology” or “fitness” OR “morbidity”)
#3	(#1 AND #2) AND language (English), See Details in Figure 1<br>Refined basis: document type: (ARTICLE) AND Time Span=2001-2021

3. Top 20 frequent keywords

The trend of 15 most frequent keywords during 2001-2021 is shown in Table S2. It can be seen that health and environment had always been hot topics in the field of air pollution and human health. Some top keywords directly refer to pollutant sources, such as air pollutants, particulate matter and ozone etc. In addition, developing countries that can be represented by China have always received a broad focus on the topic of air pollution.

Table S2 The trend of 15 most frequent keywords during 2001-2021

keywords	TP	01-07R(%)	08-14R(%)	15-21R(%)
health	4682	1(13.66)	1(11.41)	1(11.78)
environment	3228	2(8.78)	2(7.26)	2(5.47)
air pollutants	2208	3(6.83)	3(4.38)	3(4.59)
air pollution	2175	4(5.37)	5(3.00)	6(2.03)
particulate matter	1740	10(1.87)	4(3.57)	5(3.84)
aerosol	1702	—	7(1.73)	4(4.23)
environmental exposure	1653	—	8(1.52)	8(1.56)
air pollution indoor	1550	5(3.41)	6(2.46)	10(1.29)
concentration	1353	9(1.91)	10(1.38)	12(1.20)
mortality	1252	—	8(1.52)	13(1.23)
ozone	1051	6(2.93)	14(0.98)	11(1.24)
emit	1049	16(0.88)	16(0.88)	9(1.30)
China	948	8(1.95)	12(1.09)	16(1.01)
source	947	15(1.35)	13(1.03)	20(0.88)
industry	945	—	18(0.79)	14(1.16)

4. Methodology and Software

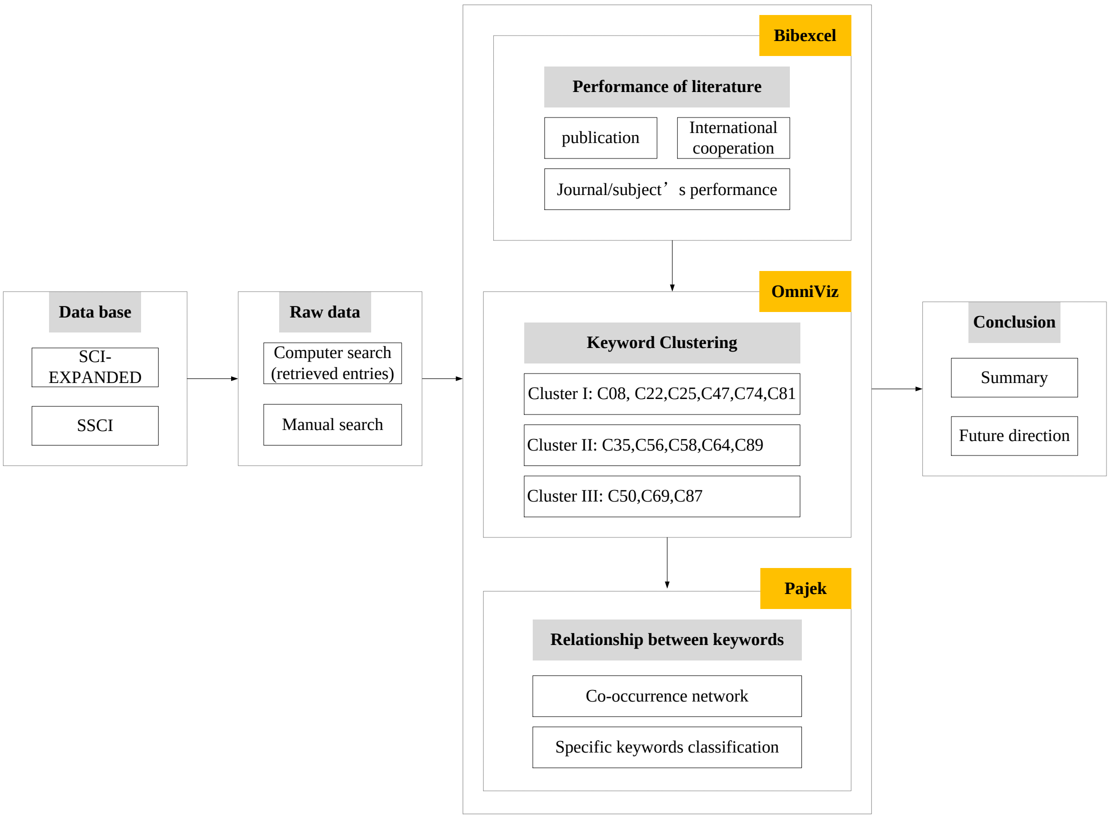

Figure S2 Methodology and software support

From Section 3.1 to Section 3.4, we use Bibexcel to analyze the literature performance in the field of air pollution and human health. BibExcel is a program dedicated to performing various types of bibliometric analyses and it is compatible with many different software suites. In Bibexcel it is possible to do most types of bibliometric analysis and allows east interaction with other software, e.g., Pajek, SPSS, Excel, etc. This method offers sufficient flexibility in both data analysis and big data management. In this paper, we first obtain review literature from the Web of Science and then use Bibexcel to deal with literature by bibliometric analysis. According to this method, we can calculate the performance of publication, international cooperation, journal's performance and subject's performance.

In Section 3.5.1, we use machine learning methods to identify the clusters of research themes via OmniViz Data Mining Software. OmniViz is a powerful data mining and analytics solution. This tool can allow us to explore critical information through mass author keywords. Utilizing sophisticated statistical and clustering algorithms can help reveal hidden patterns and features of literature performance, allowing us to discover fresh insight and analyze research areas in the field of air pollution and human health.

In Section "3.5.2 Relationship of keywords among different groups", we use this method to do social network analysis and visualize the relationship between different keywords. Pajek is used for visualization of some large networks. The main goals in the design of Pajek are to support abstraction by decomposition of a large network into smaller networks that can be treated further using more statistical methods. Moreover, it can provide us with powerful visualization results, such as Figures 8, 9

and 10. In this section, Pajek is the main tool that we used to visualize the relationship between different keywords.

5. High Co-cited Literature in Research Clusters

(1) Highly co-cited literature in cluster I

Table S3 Highly co-cited documents among research clusters in Cluster I

Cluster	Reference to co-cited document	Times cited	Degree
Air pollution source	Raaschou-Nielsen O, Andersen ZJ, Beelen R, et al. Air pollution and lung cancer incidence in 17 European cohorts: prospective analyses from the European Study of Cohorts for Air Pollution Effects (ESCAPE). Lancet Oncol.	968	76
	Guarnieri M, Balmes JR. Outdoor air pollution and asthma. Lancet.	927	73
	Guan WJ, Zheng XY, Chung KF, Zhong NS. Impact of air pollution on the burden of chronic respiratory diseases in China: time for urgent action. Lancet.	508	58
Exposure-response relationship	Burnett RT, Pope CA 3rd et al. An integrated risk function for estimating the global burden of disease attributable to ambient fine particulate matter exposure. Environ Health Perspect.	1101	82
	Chen Y, Ebenstein A, Greenstone M, Li H. Evidence on the impact of sustained exposure to air pollution on life expectancy from China's Huai River policy. Proc Natl Acad Sci U S A.	929	74
	Beelen R, Raaschou-Nielsen O, Stafoggia M, et al. Effects of long-term exposure to air pollution on natural-cause mortality: an analysis of 22 European cohorts within the multicentre ESCAPE project. Lancet.	865	68
Health & Mortality	Lelieveld J, Evans JS, Fnais M, Giannadaki D, Pozzer A. The contribution of outdoor air pollution sources to premature mortality on a global scale. Nature.	2912	87
	Cohen AJ, Brauer M, Burnett R, et al. Estimates and 25-year trends of the global burden of disease attributable to ambient air pollution: an analysis of data from the Global Burden of Diseases Study 2015. Lancet.	2800	86
	Kan H, Chen R, Tong S. Ambient air pollution, climate change, and population health in China. Environ Int.	528	60
Cost&Benefit	Sharma S, Zhang M, Anshika, Gao J, Zhang H, Kota SH. Effect of restricted emissions during COVID-19 on air quality in India. Sci Total Environ.	573	64
	Heal MR, Kumar P, Harrison RM. Particles, air quality, policy and health. Chem Soc Rev.	454	50
	Ding D, Xing J, Wang S, Liu K, Hao J. Estimated Contributions of Emissions Controls, Meteorological Factors, Population Growth, and Changes in Baseline Mortality to Reductions in Ambient PM2.5 and PM2.5-Related Mortality in China, 2013-2017. Environ Health Perspect.	125	36

(2) Highly co-cited literature in cluster II

Table S4 Highly co-cited documents among research clusters in Cluster II

Cluster	Reference to co-cited document	Times cited	Degree
Air pollution source	Cassee FR, Héroux ME, Gerlofs-Nijland ME, Kelly FJ. Particulate matter beyond mass: recent health evidence on the role of fractions, chemical constituents and sources of emission. Inhal Toxicol.	283	43
	Timmers, Victor, Peter Achten. "Non-exhaust PM emissions from electric vehicles." Atmospheric Environment.	191	38
	Pope CA 3rd, Coleman N, Pond ZA, Burnett RT. Fine particulate air pollution and human mortality: 25+ years of cohort studies. Environ Res.	113	30
Air pollution monitoring	Eeftens M, Beelen R, de Hoogh K, et al. Development of Land Use Regression models for $PM_{2.5}$, $PM_{2.5}$ absorbance, PM_{10} in 20 European study areas; results of the ESCAPE project. Environ Sci Technol.	618	68
	Brauer M, Amann M, Burnett RT, Cohen A, et al. Exposure assessment for estimation of the global burden of disease attributable to outdoor air pollution. Environ Sci Technol.	498	54
	Morawska L, Thai PK, Liu X, et al. Applications of low-cost sensing technologies for air quality monitoring and exposure assessment: How far have they gone? Environ Int.	282	42
Particulate matter concentration	Brauer M, Freedman G, Frostad J, et al. Ambient Air Pollution Exposure Estimation for the Global Burden of Disease 2013. Environ Sci Technol.	706	60
	Abhijith, K. V. et al. "Air pollution abatement performances of green infrastructure in open road and built-up street canyon environments – A review." Atmospheric Environment.	379	43
	Ghio AJ, Carraway MS, Madden MC. Composition of air pollution particles and oxidative stress in cells, tissues, and living systems. J Toxicol Environ Health B Crit Rev.	343	40
Atmospheric aerosol	Shiraiwa M, Ueda K, Pozzer A, et al. Aerosol Health Effects from Molecular to Global Scales. Environ Sci Technol.	233	39
	Stafoggia M, Bellander T, Bucci S, et al. Estimation of daily PM_{10} and $PM_{2.5}$ concentrations in Italy, 2013-2015, using a spatiotemporal land-use random-forest model. Environ Int.	155	39
	Walser SM, Gerstner DG, Brenner B, et al. Evaluation of exposure-response relationships for health effects of microbial bioaerosols - A systematic review. Int J Hyg Environ Health.	135	37

(3) Highly co-cited literature in cluster III

Table S5 Highly co-cited documents among research clusters in Cluster III

Cluster	Reference to co-cited document	Times cited	Degree
Atmospheric physics	Guttikunda, SK. and Puja Jawahar. "Atmospheric emissions and pollution from the coal-fired thermal power plants in India." Atmospheric Environment.	241	41
	Shindell, DT. and Christopher JS. "Climate and air-quality benefits of a realistic phase-out of fossil fuels." Nature.	131	35
	Sun Y, Lei L, Zhou W, et al. A chemical cocktail during the COVID-19 outbreak in Beijing, China: Insights from six-year aerosol particle composition measurements during the Chinese New Year holiday. Sci Total Environ.	94	27
	Cassee FR, Héroux ME, Gerlofs-Nijland ME, Kelly FJ. Particulate matter beyond mass: recent health evidence on the role of fractions,	283	44

	chemical constituents and sources of emission. Inhal Toxicol.		
Atmospheric chemistry	Guttikunda, SK, Puja J. “Atmospheric emissions and pollution from the coal-fired thermal power plants in India.” Atmospheric Environ.	241	40
	Sun Y, Lei L, Zhou W, Chen C, et al. A chemical cocktail during the COVID-19 outbreak in Beijing, China: Insights from six-year aerosol particle composition measurements during the Chinese New Year holiday. Sci Total Environ.	94	26
Health & Mortality	Kelly FJ, Fussell JC. Air pollution and public health: emerging hazards and improved understanding of risk. Environ Geochem Health.	618	67
	Hu X, Zhang, Y, Ding ZH et al. “Bioaccessibility and health risk of arsenic and heavy metals (Cd, Co, Cr, Cu, Ni, Pb, Zn and Mn) in TSP and PM2.5 in Nanjing, China.” Atmospheric Environment.	464	52
	Kelly FJ, Fussell JC. Air pollution and public health: emerging hazards and improved understanding of risk. Environ Geochem Health.	330	38
Public & Occupational Health	Rohde RA, Muller RA. Air Pollution in China: Mapping of Concentrations and Sources. PLoS One.	474	51
	Song C, Wu L, Xie Y, He J, Chen X, Wang T, Lin Y, Jin T, Wang A, Liu Y, Dai Q, Liu B, Wang YN, Mao H. Air pollution in China: Status and spatiotemporal variations. Environ Pollut.	412	46
	Yu Z, Shuai C, Bian J, et al. “Socioeconomic factors of PM2.5 concentrations in 152 Chinese cities: Decomposition analysis using LMDI.” J. Clean. Prod.	104	30
